# Disk compression of *k*-mer sets

**DOI:** 10.1186/s13015-021-00192-7

**Published:** 2021-06-21

**Authors:** Amatur Rahman, Rayan Chikhi, Paul Medvedev

**Affiliations:** 1 Penn State University, State College, PA USA; 2 Department of Computational Biology, C3BI USR 3756 CNRS, Institut Pasteur, Paris, France

**Keywords:** De Bruijn graphs, Compression, k-mer sets, Spectrum-preserving string sets

## Abstract

K-mer based methods have become prevalent in many areas of bioinformatics. In applications such as database search, they often work with large multi-terabyte-sized datasets. Storing such large datasets is a detriment to tool developers, tool users, and reproducibility efforts. General purpose compressors like gzip, or those designed for read data, are sub-optimal because they do not take into account the specific redundancy pattern in k-mer sets. In our earlier work (Rahman and Medvedev, RECOMB 2020), we presented an algorithm UST-Compress that uses a spectrum-preserving string set representation to compress a set of k-mers to disk. In this paper, we present two improved methods for disk compression of k-mer sets, called ESS-Compress and ESS-Tip-Compress. They use a more relaxed notion of string set representation to further remove redundancy from the representation of UST-Compress. We explore their behavior both theoretically and on real data. We show that they improve the compression sizes achieved by UST-Compress by up to 27 percent, across a breadth of datasets. We also derive lower bounds on how well this type of compression strategy can hope to do.

## Introduction

Many of today’s bioinformatics analyses are powered by tools that are $k$-mer based. These tools first reduce the input sequence data, which may be of various lengths and type, to a set of short fixed length strings called $k$-mers. *K*-mer based methods are used in a broad range of applications, including genome assembly [1], metagenomics [2], genotyping [3, 4], variant calling [5], and phylogenomics [6]. They have also become the basis of a recent wave of database search tools [7–15], surveyed in [16]. *K*-mer based methods are not new, but only recently they have started to be applied to terabyte-sized datasets. For example, the dataset used to test the BIGSI database search index, which is composed of 31-mers from 450,000 microbial genomes [11], takes about 12 TB to store in compressed form.

Storing such large datasets is a detriment to tool developers, tool users, and reproducibility efforts. For tool developers, development time is significantly increased when having to manage such large files. Due to the iterative nature of the development process, these files do not typically just sit in one place, but instead get created/moved/recreated many times. For tool users, the time it takes for the tools to write these files to disk and load them into memory is non-negligible. In addition, as we scale to even larger datasets, storage costs start to play a larger factor. Finally, for reproducibility efforts, storing and moving terabytes of data across networks can be detrimental.

To minimize these negative effects, disk compression of $k$-mer sets is a natural solution. By disk compression, we refer to a compressed representation that, while supporting decompression, does not support any other querying of the compressed data. Compressed representations that allow for membership queries [17] are important in their own right, but are sub-optimal when only storage is required. Most $k$-mer sets are currently stored on disk in one of two ways. In the situation where the set of $k$-mers comes from $k$-mer counting reads, one can simply compress the reads themselves using one of many read compression tools [18–20]. This approach requires the substantial overhead of running a $k$-mer counter as part of decompression, but it is often used in the absence of better options. The second approach is to gzip/bzip the output of the $k$-mer counter [21–25]. As we showed in [26], both of these approaches are space-inefficient by at least an order-of-magnitude. This is not surprising, as neither of these approaches was designed specifically for disk compression of $k$-mer sets.

Disk compression tailor-made for $k$-mer sets was first considered in our earlier work [26]. The idea was based on the concept of *spectrum-preserving string sets (SPSS)*, introduced in [26–28]. In [28], the concept of SPSS is introduced under the name *simplitigs*. A set of strings *S* is said to be a SPSS representation of a set of $k$-mers *K* iff 1) the set of $k$-mers contained in *S* is exactly *K*, 2) *S* does not contain duplicate $k$-mers, and 3) each string in *S* is of length $\ge k$. The weight of an SPSS is the number of characters it contains. For example, if $K = \{ACG, CGT, CGA\}$, then $\{ACGT, CGA\}$ would be an SPSS of weight 7; also *K* itself would be an SPSS of *K* of weight 9. On the other hand, $\{CGACGT\}$ is not an SPSS, because it contains $GAC \notin K$. Intuitively, a low weight SPSS can be constructed by gluing together $k$-mers in *K*, with each glue operation reducing the weight by $k-1$. In [26], we proposed the following simple compression strategy, called UST-Compress. We find a low-weight SPSS *S*, using a greedy algorithm called UST, and compress *S* to disk using a generic nucleotide compression algorithm (e.g. MFC [29]). UST-Compress achieved significantly better compression sizes than the two approaches mentioned above.

UST-Compress was not designed to be the best possible disk compression algorithm but only to demonstrate one of the possible applications of the SPSS concept. When the goal is specifically disk compression, we are no longer bound to store a set of strings with exactly the same $k$-mers as *K*, as long as a decompression algorithm can correctly recover *K*. The main idea of this paper is to replace the SPSS with a more relaxed string set representation, over the alphabet $\{A,C,G,T,[,],+,-\}$. Our approach is loosely inspired by the notion of elastic-degenerate strings [30]. It attempts to remove even more duplicate $(k-1)$-mers from the representation than SPSS does, using the extra alphabet characters as placeholders for nearby repetitive $(k-1)$-mers. For the above example, our representation would be $ACG[+A]T$, where the $``+''$ is interpreted as a placeholder for the $k-1$ characters before the open bracket (i.e. *CG*). After replacing the $``+''$, we get *ACG*[*CGA*]*T* and we split the string by cleaving out the substring within brackets; i.e., we get *ACGT* and *CGA*.

Based on this idea, we present two algorithms for the disk compression of $k$-mer sets, ESS-Compress and ESS-Tip-Compress. We explore the behavior of these algorithms both theoretically and on real data. We give a lower bound on how well this type of algorithm can compress. We show that they improve the compression sizes achieved by UST-Compress by up to 27% across a breadth of datasets. The two algorithms present a trade-off between time/memory and compression size, which we explore in our results. The two algorithms are freely available open source tools on http://github.com/medvedevgroup/ESSCompress.

## Preliminaries

### Basic definitions

#### Strings

The length of string *x* is denoted by |*x*|. A string of length *k* is called a *k-mer*. We assume $k$-mers are over the DNA alphabet. A string over the alphabet $\{A,C,G,T,[,],+,-\}$ is said to be *enriched*. We use $\cdot$ as the string concatenation operator. For a set of strings *S*, $weight(S) = \sum _{ x \in S} |x|$ denotes the total count of characters. We define $suf_k(x)$ (respectively, $pre_k(x)$) to be the last (respectively, first) *k* characters of *x*. We define $cutPre_{k}(x) = suf_{|x| - k}(x)$ as *x* with the prefix removed. When the subscript is omitted from *pre*, *suf*, and *cutPre*, we assume it is $k-1$. A string *x* is *canonical* if it is the lexicographically smaller of *x* and its reverse complement.

For *x* and *y* with $suf(x) = pre(y)$, we define *gluing* *x* and *y* as $x \odot y = x\cdot cutPre(y)$. For $s \in \{0, 1\}$, we define *orient*(*x*, *s*) to be *x* if $s=0$ and to be the reverse complement of *x* if $s=1$. We say that $x_0$ and $x_1$ have a $(s_0, s_1)$-*oriented-overlap* if $suf(orient(x_0, 1 - s_0)) = pre(orient(x_1, s_1))$. Intuitively, such an overlap exists between two strings if we can orient them in such a way that they are glueable. For example, *AAC* and *TTG* have a (0, 0)-oriented overlap.

#### Bidirected de Bruijn graphs

**Fig. 1:**
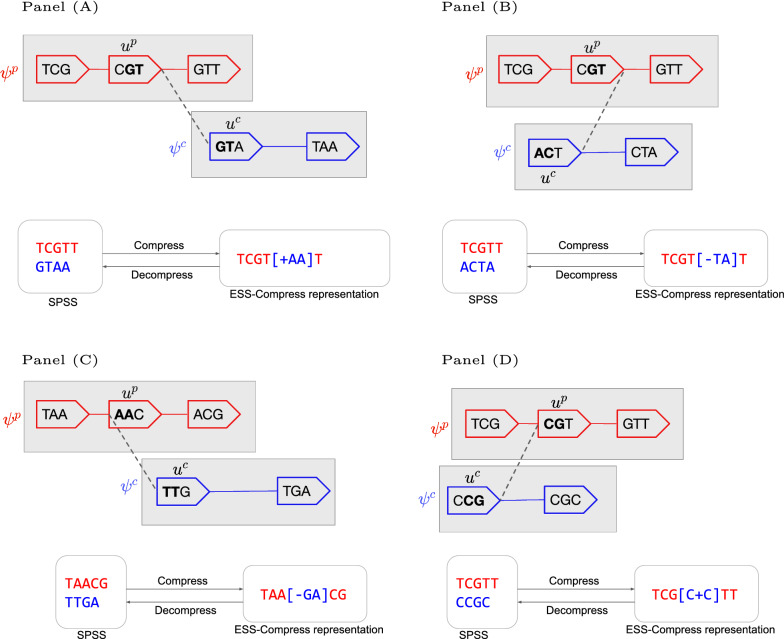
Examples of the four types of absorption. Each panel shows the edges along two paths: $\psi ^p$ (red vertices inside a shaded rectangle) and $\psi ^c$ (blue vertices inside a shaded rectangle) and an absorption edge $e=\{(u^p, s^p),(u^c,s^c)\}$ (dashed line) between the parent unitig $u^p$ and the child unitig $u^c$. The graph being shown in each panel is *cdBG*(*K*), but only the absorption edge and the edges of $\psi ^p$ and $\psi ^c$ are shown. In this simple example, the unitigs of *dBG*(*K*) are just paths made of single vertices, and hence the vertices of *cdBG*(*K*) have labels of length $k=3$. Each vertex is shown as a pointed rectangle with its label inside; we use the convention that the “zero” side of a vertex is the flat side on the left, and the “one” side is the pointy side on the right. At the bottom left of each panel, we show the spectrum-preserving string set (SPSS) $spell(\{\psi ^p, \psi ^c\})$. At the bottom right, we show the enriched representation generated by our algorithm. Depending on the value of $s^p$ and $s^c$, four different cases can arise. When $s^p = 1, s^c = 0$ (shown in **A**), $pre(lab(u^c))$ is replaced with marker “$+$”, as it is same as $suf(lab(u^p))$. When $s^p = 1, s^c = 1$ (shown in **B**), $pre(lab(u^c))$ is replaced by “−”, as it is same as the reverse complement of $suf(lab(u^p))$. When $s^p = 0, s^c = 0$ (shown in **C**), $pre(lab(u^c))$ is replaced with “−”, as it is the same as the reverse complement of $pre(lab(u^p))$. When $s^p = 0, s^c = 1$ (shown in **D**), $suf(lab(u^c))$ is replaced with “$+$”, as it is the same as $pre(lab(u^p))$

A bidirected graph *G* is a pair (*V*, *E*) where the set *V* are called vertices and *E* is a set of edges. An edge *e* is a set of two pairs, $\{(u_0, s_0), (u_1, s_1)\}$, where $u_i \in V$ and $s_i \in \{0, 1\}$, for $i \in \{0, 1\}$. Note that this differs from the notion of an edge in an undirected graph, where $E \subseteq V \times V$. Intuitively, every vertex has two sides, and an edge connects to a side of a vertex (see Fig. 1 for examples). An edge is a *loop* if $u_0 = u_1$. Given a non-loop edge *e* that is incident to a vertex *u*, we denote *side*(*u*, *e*) as the side of *u* to which it is incident. We say that a vertex *u* is a *dead-end* if it has exactly one side to which no edges are incident. A *bidirected DNA graph* is a bidirected graph *G* where every vertex *u* has a string label *lab*(*u*), and for every edge $e = \{(u_0, s_0), (u_1, s_1)\}$, there is a $(s_0, s_1)$-oriented-overlap between $lab(u_0)$ and $lab(u_1)$ (see Fig. 1 for examples). *G* is said to be *overlap-closed* if there is an edge for every such overlap. Let *K* be a set of canonical $k$-mers. The node-centric *bidirected de Bruijn graph*, denoted by *dBG*(*K*), is the overlap-closed bidirected DNA graph where the vertices and their labels correspond to *K*. In this paper, we will assume that *dBG*(*K*) is not just a single cycle; such a case is easy to handle in practice but is a space-consuming corner-case in all the analyses.

#### Paths and spellings

A sequence $p = (u_0, e_1, u_1, \ldots , e_n, u_n)$ is a *path* iff (1) for all $1 \le i \le n$, $e_i$ is incident to $u_{i-1}$ and to $u_{i}$, (2) for all $1 \le i \le n - 1$, $side(u_i, e_i) = 1 - side(u_i, e_{i+1})$, and (3) all the $u_i$s are different. A path can also be any single vertex. Vertices $u_1, \ldots , u_{n-1}$ are called *internal* and $u_0$ and $u_n$ are called *endpoints*. We call $u_0$ to be the *initiator* vertex of *p*. We say that *p* is *normalized* if for every $e_i$, $side(u_{i-1},e_i) = 1$ and $side(u_i, e_i) = 0$; intuitively, the path uses edges like in a directed graph. The *spelling* of a normalized path *p* is defined as $spell(p) = lab(u_0) \odot \cdots \odot lab(u_n)$. If *P* is a set of normalized paths, then $spell(P) = \bigcup _{p \in P} spell(p)$.

#### Unitigs and the compacted de Bruijn graph

A path in *dbG*(*K*) is a *unitig* if all its vertices have in- and out-degrees of 1, except that the first vertex can have any in-degree and the last vertex can have any out-degree. A single vertex is also a unitig. A unitig is *maximal* if it is not a sub-path of another unitig. It was shown in [31] that if *dBG*(*K*) is not a cycle, then the set of maximal unitigs forms a unique decomposition of the vertices in *dBG*(*K*) into vertex-disjoint paths. The bidirected *compacted de Bruijn graph* of *K*, denoted by *cdBG*(*K*), is the overlap-closed bidirected DNA graph where the vertices are the maximal unitigs of *dBG*(*K*), and the labels of the vertices are the spellings of the unitigs. In practice, this graph can be efficiently constructed from *K* using the BCALM2 tool [31, 32].

#### Spanning out-forest

Given a directed graph *D*, an *out-tree* is a subgraph in which every vertex except one, called the root, has in-degree one, and, when the directions on the edges are ignored, is a tree. An *out-forest* is a collection of vertex-disjoint out-trees. An out-forest is *spanning* if it covers all the vertices of *D*.

### Path covers and UST-compress

**Fig. 2:**
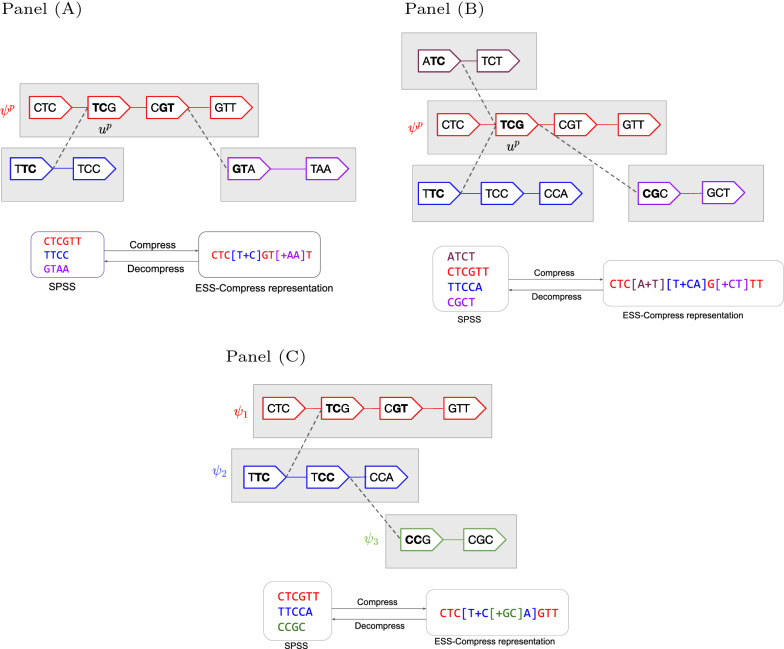
More complex absorption examples. In **A**, one path absorbs multiple paths. In **B**, one unitig $u^p$ absorbs multiple paths. In **C**, one path ($\psi _1$) absorbs another ($\psi _2$) which itself absorbs another ($\psi _3$). This is a recursive absorption, showing how a path can be both a child and a parent

A *vertex-disjoint normalized path cover* $\Psi$ of *cdBG*(*K*) is a set of normalized paths such that every vertex is in exactly one path and no path visits a vertex more than once; we will sometimes use the shorter term *path cover* to mean the same thing. There is a close relationship between SPSS representations of *K* and path covers, shown in [26]. In particular, a path cover $\Psi$ induces the SPSS $spell(\Psi )$. An example of a path cover is one where every vertex of *cdBG*(*K*) is in its own path, and the corresponding SPSS is the set of all maximal unitig sequences. Figures 1 and 2 show examples of path covers. The number of paths in $\Psi$ (denoted as $|\Psi |$) and the weight of the induced SPSS is closely related:

2.1 $$\begin{aligned} weight(spell(\Psi )) = |K| + |\Psi |(k-1) \end{aligned}$$

This relationship also translates to the number of edges in $\Psi$; by its definition, the number of edges in $\Psi$ is simply the number of vertices in *cdBG*(*K*) minus $|\Psi |$.

The idea of our previous algorithm UST-Compress [26] is to find a path cover $\Psi _{UST}$ with as many edges as possible. Having more edges reduces the number of paths, which in turn reduces the weight of the corresponding SPSS and the size of the final compressed output. We can understand this intuitively as follows. Edges in *cdBG*(*K*) connect unitigs whose endpoints have the same $(k-1)$-mer (after accounting for reverse complements). For every edge we add to our path cover, we glue these two unitigs and remove one duplicate instance of the $(k-1)$-mer from the corresponding SPSS. Note however that $\Psi _{UST}$ does not remove all duplicate $(k-1)$-mers from the SPSS, because $\Psi$ can only have two edges incident on a vertex, one from each side, and hence a unitig can only be glued at most twice. If a unitig has edges to more than two other unitigs, then some of the adjacent unitigs would include the duplicate $(k-1)$-mer in the SPSS. The idea of our paper is to exploit the redundancy due to those remaining edges an thus further reduce the size of the representation.

## ESS-compress

### Main algorithm

Our starting point is a set of canonical $k$-mers *K*, the graph *cdBG*(*K*), and a vertex-disjoint normalized path cover $\Psi$ of *cdBG*(*K*) returned by UST.^1^ To develop the intuition for our algorithm, we first consider a simple example (Fig. 1A). In this example, we see a vertex-disjoint path cover $\Psi$ composed of two paths, $\psi ^p$ and $\psi ^c$. There is an edge between an internal vertex (=unitig^2^) $u^p$ of $\psi ^p$ and the initiator vertex $u^c$ of $\psi ^c$. Such an edge is an example of an absorption edge. ESS-Compress constructs an enriched string representation of *K*, as shown in the figure. The basic idea is that $u^p$ and $u^c$ share a common $(k-1)$-mer (i.e. *GT*). We can cut out this common portion from the string representing $u^c$ and replace it with a special marker character “+”. We can then include $u^c$ inside of the representation of $u^p$ by surrounding $u^c$ with brackets. The marker character $``+''$ is a placeholder for the $k-1$ nucleotides right before the opening bracket. To decompress the enriched string, we first replace the marker to get *TCGT*[*GTAA*]*T* and then cleave out the bracketed string to get $\{TCGTT, GTAA\}$. This exactly recovers the SPSS representation of $\psi ^p$ and $\psi ^c$.

Formally, we say that an edge in *cdBG*(*K*) is an *absorption edge* iff (1) it connects two unitigs $u^p$ and $u^c$, on two distinct paths $\psi ^p$ and $\psi ^c$, respectively, (2) $u^p$ is an internal vertex, and (3) $u^c$ is an initiator vertex. We refer to $u^p$ and $\psi ^p$ as *parents* and $u^c$ and $\psi ^c$ as *children*; we also say that $\psi ^p$ and $u^p$ absorb $\psi ^c$ and $u^c$.^3^

Figure 1B–D shows the other cases, corresponding to the possible orientation of the absorption edge. The logic is the same, but we need to introduce a second marker character $``-''$ that is a placeholder for the reverse complement of the last $k-1$ characters right before the opening bracket. In each of these cases, we add 3 extra characters (two brackets and one marker) and remove $k - 1$ nucleotide characters. Note that, expanding the alphabet has its inherent cost, but even after taking that into account, we get lower number of characters than SPSS representation when $k > 4$.

Next, observe that a single parent path can absorb multiple children paths, as illustrated in Fig. 2A. Also, observe that a single parent unitig can absorb more than one child path, as shown in Fig. 2B. As in the previous example, we save $k - 1 - 3 = k -4$ characters for every absorbed edge.

These absorptions can be recursively combined, as shown in Fig. 2C. Because we require a parent unitig to be an internal vertex and a child unitig to be an initiator vertex, the same unitig cannot be both parent and child. Therefore, ESS-Compress can construct a representation recursively, without any conflicts. The recursion tree is reflected in the nesting structure of the brackets in the enriched string.

However, we must be careful to avoid cycles in the recursion. We define the *absorption digraph* $D_A$ as the directed graph whose vertex set is the set of paths $\Psi$ and an edge $(\psi ^p \rightarrow \psi ^c)$ if $\psi ^p$ absorbs $\psi ^c$. For every edge in $D_A$, we also associate the corresponding bidirected edge between $u^p$ and $u^c$ in *cdBG*(*K*). We would like to select a subset of edges *F* along which to perform absorptions, so as to avoid cycles in $D_A$ and to make sure a path cannot be absorbed by more than one other path. We would also try to choose as many edges as possible, since each absorption saves $k-4$ characters. To achieve these goals, ESS-Compress defines *F* as a spanning out-forest in $D_A$ with the maximum number of edges. We postpone the algorithm to find *F* to Sect. "Algorithm to choose absorption edges".

**Fig. 3:**
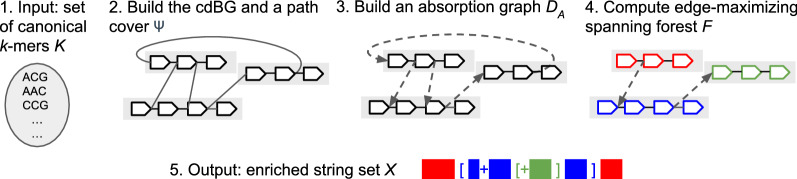
Visual overview of the steps in Algorithm 1

The high-level pseudo-code of ESS-Compress is shown in Algorithm 1 and illustrated in Fig. 3. The recursive algorithm to create the enriched representation using *F* as a guide is shown in Algorithm 2. It follows the intuition we just developed. It starts from the paths that will not be absorbed (i.e. the roots in *F*). For a path $\psi ^p$, it first computes the enriched representations of all the child paths (Lines 3–9). It then integrates them into the appropriate locations of $spell(\psi ^p)$ (Lines 10–14). It then uses a marker to replace the redundant sequence in the spelling of $\psi ^p$, with respect to $\psi ^p$’s own parent (Lines 17–31). To decide which marker to use, it receives as a parameter the absorption edge $e_D$ that was used to absorb $\psi ^p$.

Decompression is done by a recursive algorithm DEC that takes as input an enriched string *x* and a $(k-1)$-mer called *markerReplacement*. Initially, DEC is called independently on every enriched string $x\in \text {ESS-Compress} (K)$, with $markerReplacement = null$. We call the characters of *x* which are not enclosed within brackets *outer*. The brackets themselves are not considered outer characters. DEC first replaces any occurrence of an outer $``+''$ (respectively, $``-''$) with *markerReplacement* (respectively, the reverse complement of *markerReplacement*). It then outputs all the outer characters as a single string. Then, for every top-level open/close bracket pair in *x*, it calls DEC recursively on the sequence in between the brackets, and passes as *markerReplacement* the rightmost $k-1$ outer characters to the left of the open bracket.

### Algorithm to choose absorption edges

Let *D* be any directed graph and consider the problem of finding a spanning out-forest with the maximum number of edges. We call this the problem of finding an *edge-maximizing spanning out-forest*. This problem is a specific instance of the maximum weight out-forest problem [33], which allows for weights to be placed on the edges. As we show in this section, there is an optimal algorithm for our problem that is simpler than the algorithm for arbitrary weights described in [33].
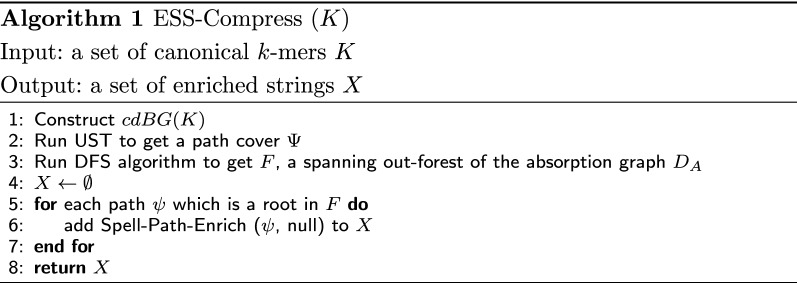


Our algorithm first decomposes *D* into strongly connected components, and builds *SC*(*D*), the strongly connected component digraph of *D*. In *SC*(*D*), the vertices are the strongly connected components of *D*, and there is an edge from component $c_1$ to $c_2$ if there is an edge in *D* from some vertex in $c_1$ to some vertex in $c_2$. For every component that is a source in *SC*(*D*), we pick an arbitrary vertex from it (in *D*) and put it into a “starter” set. Then, we perform a depth-first search (DFS) traversal of *D*, but whenever we start a new tree, we initiate it with a vertex from the starter set, if one is available. We remove the vertex from the starter set once it is used to initiate a tree. We then output the DFS forest *F*.

We will prove that *F* is a spanning out-forest of *D* with the maximum number of edges.

#### Lemma 3.1

(*Correctness of edge-maximizing spanning out-forest algorithm*) *Let* *D* *be a directed graph, let* *F* *be the spanning out-forest returned by our algorithm run on* *D*, and let $n_{sc}$ *be the number of source components in* *SC*(*D*). *Then, the number of out-trees in* *F* is $n_{sc}$ *and this is the smallest possible for any spanning out-forest. Also, the number of edges in* *F* *is the maximum possible for any spanning out-forest*.

#### *Proof*

Consider any spanning out-forest of *D*. If it has less than $n_{sc}$ out-trees, then by the pigeonhole principle, there are two source components $c_1$ and $c_2$ with vertices $v_1$ and $v_2$, respectively, belonging to the same out-tree. This is a contradiction, since $c_1$ and $c_2$ are source components and hence there cannot be a path between them. Hence, any spanning out-forest must have at least $n_{sc}$ out-trees. Now, consider *F*. Every vertex in *D* is reachable from one of the vertices in the starter set, by its construction. There are $n_{sc}$ starter vertices, so *F* will have at most $n_{sc}$ out-trees. Since any spanning out-forest must have at least $n_{sc}$ out-trees, *F* will have $n_{sc}$ out-trees and it will be the minimum achievable. Also, in any spanning out-forest, the number of edges is the number of vertices minus the number of out-trees; hence *F* will have the the maximum number of edges of any spanning out-forest. $\square$

### The weight of the ESS-compress representation

In this section, we derive a formula for the weight of the ESS-Compress representation and explore the potential benefits of some variations of ESS-Compress.
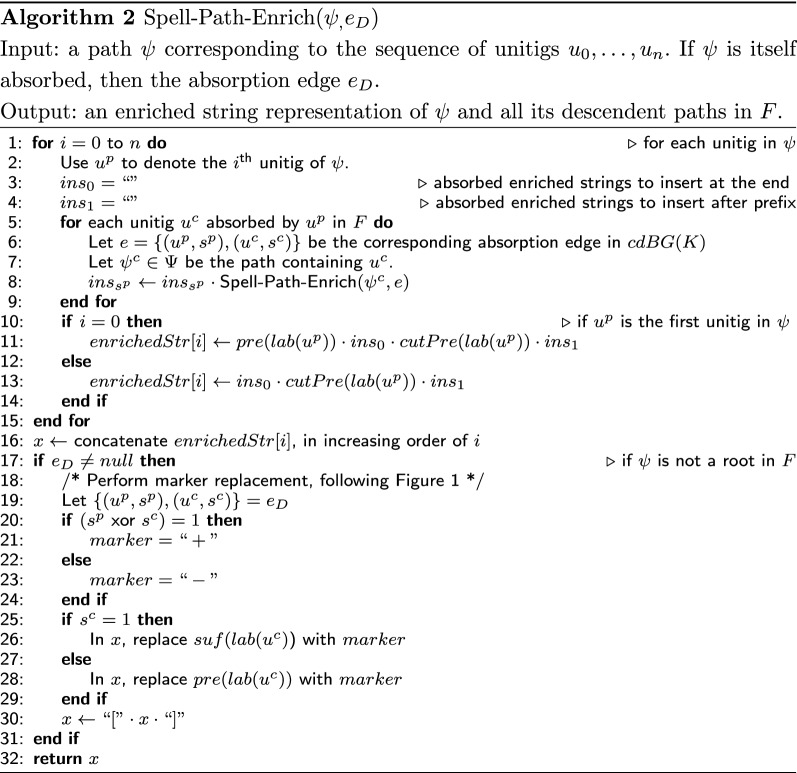


#### Theorem 3.2

Let *K* be a set of canonical $k$-mers, and let $\Psi$ be a vertex-disjoint normalized path cover of *cdBG*(*K*) that is used by $\text {ESS-Compress} (K)$. Let $n_{sc}$ be the number of sources in the strongly connected component graph of the absorption graph $D_A$. Let *X* be the solution returned by $\text {ESS-Compress} (K)$. Then

$$\begin{aligned} weight(X) = |K| + 3|\Psi | + n_{sc} (k-4) \end{aligned}$$

#### *Proof*

If we unroll the recursion of ESS-Compress, then there are exactly $|\Psi |$ runs of Spell-Path-Enrich, one for each $\psi \in \Psi$. For each call, we let $n_\psi$ be the number of characters in the returned string that are added non-recursively (i.e. everything except $ins_0$ and $ins_1$). Considering the structure of the recursion and accounting for characters in this way, we have that $weight(X) = \sum _{\psi \in \Psi } n_\psi$.

Prior to marker replacement (Line 17, the non-recursive part of *x* is $spell(\psi )$). When $\psi$ is a root in the absorption forest *F*, then the marker absorption stage is not executed and so $n_\psi = |spell(\psi )|$. Otherwise, the marker absorption phase (Lines 17 to 31) removes $k-1$ characters, adds 1 new marker character, and adds two new bracket characters. Hence, $n_\psi = |spell(\psi )| - (k-1) + 3 = |spell(\psi )| - (k - 4)$. By Lemma 3.1, *F* contains $n_{sc}$ roots. Hence,

$$\begin{aligned} weight(X)&= \sum _{\psi \in \Psi } n_\psi = \sum _{\psi \text { is a root}} |spell(\psi )| + \sum _{\psi \text { is not a root}} \left( |spell(\psi )| - (k-4) \right) \\&= \sum _{\psi \in \Psi } |spell(\psi )| - (k-4)(|\Psi | - n_{sc}) = |K| + 3|\Psi | - n_{sc} (k-4) \end{aligned}$$

The last equality follows by applying Eq. (2.1) from Sect. "Preliminaries". $\square$

We can use Theorem 3.2 to better understand ESS-Compress. The weight depends on the choice of $\Psi$. The $\Psi$ returned by UST has, empirically, almost the minimum $|\Psi |$ possible [26]. This (almost) minimizes the $3|\Psi |$ term in Theorem 3.2. However, this may not necessarily lead to the lowest total weight, because there is an interplay between $\Psi$ and $n_{sc}$, as follows. Let $\Psi '$ be a vertex-disjoint normalized path cover with $|\Psi '| > |\Psi |$. Its paths are shorter, on average, than $\Psi$’s. There may now be edges of *cdBG*(*K*) that become absorption edges, that were not with $\Psi$. For example, an edge between two unitigs which are internal in $\Psi$ is not, by our definition, an absorption edge. With the shorter paths in $\Psi '$, one of these unitigs may become an initiator vertex, making the edge absorbing. This may in turn improve connectivity in $D_A$ and decrease $n_{sc}$, counterbalancing the increase in $|\Psi '|$. Nevertheless, ESS-Compress does not consider alternative path covers and always uses the one returned by UST.

Another aspect of ESS-Compress that could be changed is the definition of absorption edge. We restrict absorption edges to be between an initiator unitig and an internal unitig; however, one could in principle also define ways to absorb between an endpoint unitig and an internal unitig, or between two internal unitigs. This could potentially decrease $n_{sc}$ by increasing the number of absorption edges, though it would likely need more complicated and space-consuming encoding schemes.

How much could be gained by modifying the path cover and the absorption rules that ESS-Compress uses? We can answer this by observing that $n_{sc}$ cannot be less than *C*, the number of connected components of the undirected graph underlying *cdBG*(*K*). At the same time, in [26] we gave an algorithm to compute an instance-specific lower bound $\beta$ on the number of paths in any vertex-disjoint path cover. Putting this together, we conclude that regardless of which path cover is used and which subset of *cdBG*(*K*) edges are allowed to be absorbing, the weight of a ESS-Compress representation cannot be lower than:

3.1 $$\begin{aligned} |K| + 3\beta + C(k-4) \end{aligned}$$

As we will see in the results, the weight of ESS-Compress is never more than 2% higher than this lower bound, which is why we did not pursue these other possible optimizations to ESS-Compress. We note, however, that the above is not a general lower bound and does not rule out the possibility of lower-weight string set representations that beat ESS-Compress.

## ESS-Tip-compress: a simpler alternative

ESS-Compress is designed to achieve a low compression size but can require a large memory stack due to its recursive structure. The memory during compression and decompression is proportional to the depth of this stack, which is the depth of the out-forest *F*. If *F* were to be more shallow, then the memory would be reduced. In this section, we describe ESS-Tip-Compress, a simpler, faster, and lower-memory technique that can be used when compression speed/memory are prioritized. It is centered on dead-end vertices in the compacted graph, which usually correspond to tips in the uncompacted dBG and are typically due to sequencing errors, endpoints of transcripts, or coverage gaps. ESS-Tip-Compress is based on the observation that a large chunk of the graph is dead-end vertices (at least for sequencing data), and limiting absorption to only them can yield much of the benefits of a more sophisticated algorithm.

First, we find a vertex-disjoint normalized path cover $\Psi$ that is forced to have each dead-end vertex in its own dedicated path (i.e. its path only contains the vertex itself). This can be done easily by running UST on the graph obtained from *cdBG*(*K*) by removing all dead-end vertices. Next, we select the absorption forest *F* as follows. For each dead-end vertex *v*, we identify a non-dead-end vertex *u* which is connected to *v* via an edge *e*. In the rare case that such a *u* does not exist, we skip *v*. Otherwise, we add $(u \rightarrow v)$ to *F*. We can assume without loss of generality that $side(u,e) = 1 - side(v,e)$ because if that is not the case, than we can replace *lab*(*v*) by its reverse complement and thereby change the side to which *e* is incident. For any paths that remain uncovered by *F*, we add them as roots of their own tree. Finally, we run a slightly modified version of Spell-Path-Enrich, using this $\Psi$ and this *F*.

We modify Spell-Path-Enrich as follows. First, observe that *F* has max depth of 2 vertices. Hence, the parenthesis generated by Spell-Path-Enrich are never nested. Second, observe that the marker value is always $``+''$, because $side(u,e) = 1- side(v,e)$ for all absorption edges in *F*. These observations allow us to reduce the number of extra characters we need for each absorption down to 2, instead of 3 (we omit the implementation details).

## Empirical results

We evaluated our methods on one small bacterial dataset, two metagenomic datasets from NIH human microbiome project, reads from whole human genome, and RNA-seq reads from both human and plant (Table 1). To obtain the set of $k$-mers *K* from these datasets, we ran the DSK $k$-mer-counter [22] with $k=31$ and filtered out low-frequency k-mers ($<5$ for whole human genome and $<2$ for the other datasets). We then constructed *cdBG*(*K*) using BCALM2. The last three columns in Table 1 show the properties of the graph: number of vertices, number of dead-end vertices and total percentage of isolated vertices. We ran all our experiments single-threaded on a server with an Intel(R) Xeon(R) CPU E5-2683 v4 @ 2.10 GHz processor with 64 cores and 512 GB of memory. We used /usr/bin/time to measure time and memory. Detailed steps to reproduce our experiments are available at https://github.com/medvedevgroup/ESSCompress/tree/master/experiments.

The output of our tools was compressed with MFC. Note that MFC is not optimized for non-nucleotide characters, but such characters are rare in our string sets ($<0.1$ bits per $k$-mer). We compared our tools against four other approaches. The first is UST-Compress, which we showed in our previous work to outperform other disk compressors [26]. The second is to strip the read FASTA files of all non-sequence information and compress them using MFC. The third is to simply write one distinct $k$-mer per line to a file and compress it using MFC. The fourth is the BOSS method, as implemented in [34]. BOSS is a succinct implementation of a de Bruijn graph [35]. Though it is designed to answer membership queries, it also achieved the closest compression size to UST-Compress in our previous study [26]. As in [26], we compressed BOSS’s binary output using LZMA. We confirmed the correctness of all evaluated tools, including our own, on the datasets.

We did not explore the possibility of replacing UST in our pipeline with ProphAsm [36]. ProphAsm is an alternative algorithm to compute an SPSS called simplitigs, but we showed in [26] that the UST SPSS representation is nearly optimal, with only 2–3% difference to the lower bound of weight. Since ProphAsm computes the same kind of representation, it is impossible for it to improve result beyond 2–3%. We also did not compare against other $k$-mer membership data structures because in our previous paper [26], we showed that UST-Compress and BOSS achieve a better compression ratio on the tested datasets.

**Table 1:** Dataset characteristics

Dataset	Source	Read length (bp)	# Reads	# Distinct 31-mers	# unitigs	% Dead-end unitigs (%)	% Isolated unitigs (%)
R. sphaeroides	GAGE [37]	101	2,050,868	5,908,467	442,681	47	8
Human RNA-seq	SRR957915	101	49,459,840	101,017,526	7,665,682	4	13
Gingiva metagenome	SRS014473	101	55,419,548	101,872,420	5,678,516	36	15
Soybean RNA-seq	SRR11458718	125	83,594,116	111,206,789	3,659,969	28	12
Tongue metagenome	SRS011086	101	81,664,789	165,159,726	11,358,233	37	11
Whole human	ERR174310	101	207,579,467	2,319,022,432	51,094,913	14	18

### String set properties

We first measure the weights and sizes of our ESS-Compress and ESS-Tip-Compress, shown in Table 2. ESS-Compress uses 13–42% less characters than UST. ESS-Tip-Compress was worse than ESS-Compress (6–13% larger), but still better than UST-Compress (3–38% smaller). The lower bound computed by Eq. (3.1) is very close to the weight of ESS-Compress (within 1.7%, Table 2), indicating that the alternate strategies explored in Sect. "The weight of the ESS-Compress representation" would not be useful on these datasets.

**Table 2:** The weights and sizes of various string set representations

Dataset	UST		ESS-Tip-Compress		ESS-Compress		Eq. (3.1) lower bound
	# strings	#char/ $k$-mer	# strings	#char/ $k$-mer	# strings	#char/ $k$-mer	#char/ $k$-mer
R. sphaeroides	240,562	2.22	61,909	1.38	36,456	1.29	1.28
Human RNA-seq	4,098,389	2.22	1,834,945	1.60	1,098,938	1.42	1.39
Gingiva metagenome	3,095,476	1.91	1,499,270	1.48	917,388	1.33	1.32
Soybean RNA-seq	1,806,078	1.49	1,137,350	1.32	515,244	1.17	1.17
Tongue metagenome	6,030,814	2.10	2,664,422	1.53	1,327,701	1.33	1.32
Whole human	22,072,219	1.32	21,320,263	1.28	10,321,275	1.15	1.14

The rightmost column shows the lower bound computed by Eq. (3.1) in Sect. "The weight of the ESS-Compress representation". The weight of ESS-Compress was verified to be the same as predicted by Theorem 3.2

### Compression size

Table 3 shows the final compression sizes, after the string sets are compressed with MFC. ESS-Compress outperforms the second best tool (which is usually UST-Compress) by 4–27%. It outperforms the naive strategies (i.e. read FASTA or one $k$-mer per line) by an order-of-magnitude. Interestingly, it outperforms ESS-Tip-Compress by only 1–8%; this can be attributed to the large number of dead-end vertices (Table 1).

We observe that our improvement in weight (Table 2) does not directly translate to improvement after compression with MFC (Table 3). For ESS-Compress, the average improvement in weight over UST is 30% but the improvement in bits is 17%. We attribute this to the fact that MFC works by exploiting redundant regions, based on their context. Thus, the redundant sequence that ESS-Compress removes is likely the sequence that was more compressible by MFC and hence MFC looses some of its effectiveness.

**Table 3:** The compression sizes, as measured in bits per $k$-mer in the compressed output

Dataset	Read FASTA	One $k$-mer per line	BOSS	UST-Compress	ESS-Tip-Compress	ESS-Compress
R. sphaeroides	45.4	28.4	6.55	3.93	2.90	2.87
Human RNA-seq	45.8	31.7	6.89	4.14	3.43	3.33
Gingiva metagenome	48.0	32.4	10.64	3.76	3.22	3.05
Soybean RNA-seq	43.0	33.1	5.97	2.83	2.66	2.55
Tongue metagenome	48.1	33.3	3.59	4.07	3.32	3.07
Whole human	31.9	48.2	4.65	2.49	2.46	2.40

All string representations (i.e. not BOSS) are compressed using MFC in the final step. Since BOSS is a binary representation, we use LZMA for the final compression step

**Fig. 4:**
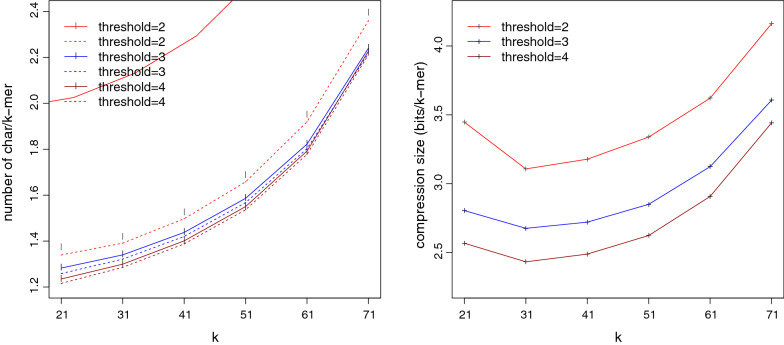
Compression performance of ESS-Compress when varying *k* and the low-frequency filter threshold, on Human RNA-seq dataset. In the left panel, solid lines represent the weight of the ESS-Compress representation, compared against the lower bound, represented by the dashed lines. In the right panel, compressed sizes are shown in bits/$k$-mer

We also verified that ESS-Compress can successfully compress datasets of varying $k$-mer sizes (between 21 and 71) and low-frequency thresholds (2, 3, and 4). Figure 4 shows compressed sizes of human RNA-seq data in bits/$k$-mer as well as their weights compared to the lower bounds. The weight of ESS-Compress closely matches the lower bound across all parameters ($<2.4\%$ gap), but the weight and compression size increase for larger *k* and lower thresholds.

### Decompression and compression time and memory

The cost of decompression is important since it is incurred every time the dataset is used for analysis. For both ESS-Compress and ESS-Tip-Compress, the decompression memory is $<1$ GB (Table 5) the time is $<10$ min for the large whole human dataset and $<1.5$ minutes for the other datasets (Table 4). Both of these are dominated by the MFC portion.

**Table 4:** Decompression time in seconds

Dataset	UST-Compress	ESS-Tip-Compress			ESS-Compress		
	MFC-D	MFC-D	Core	Total	MFC-D	Core	Total
R. sphaeroides	3	2	1	4	2	1	3
Human RNA-seq	40	41	19	60	34	17	51
Gingiva metagenome	37	38	16	54	30	15	45
Soybean	31	33	13	46	29	13	42
Tongue metagenome	62	61	28	89	49	25	74
Whole human	302	337	259	596	303	250	553

The time is broken down into the portion taken by MFC to decompress the binary file into an enriched string set and the portion taken by our core algorithm to decompress the enriched string set into an SPSS. Note that BOSS does not implement decompression (because it is a membership data structure) so it is not included

**Table 5:** Peak memory usage for compression and decompression

Dataset	Compression (GB)				Decompression (MB)				
	BOSS	UST-Compress	ESS-Tip-Compress	ESS-Compress	UST-Compress	ESS-Tip-Compress		ESS-Compress	
					MFC-D	MFC-D	Core	MFC-D	Core
R. sphaeroides	2	3	3	3	509	513	3	513	4
Human RNA-seq	4	3	3	6	515	515	3	515	38
Gingiva metagenome	4	2	2	5	515	515	3	515	4
Soybean	4	2	2	3	515	515	3	515	12
Tongue metagenome	4	2	2	9	515	515	3	515	6
Whole human	5	12	11	42	515	515	3	515	735

Decompression takes far less memory than compression, so compression memory is shown in *GB* and decompression memory in *MB*. Decompression memory is split in the same manner as the running time in Table 4

Compression is typically done only once, but the time and memory use can still be important in some applications. Tables 5 and 6 show the compression time and memory usage. For UST-Compress, the time is dominated by the *cdBG* construction step (i.e. BCALM2). For ESS-Compress, the time and memory are significantly increased beyond that. Here, the advantage of ESS-Tip-Compress stands out. Its run time is nearly the same as UST-Compress, and its memory, while higher than UST-Compress, is significantly lower than ESS-Compress.

**Table 6:** Compression time, measured in minutes

Dataset	BOSS	BCALM	UST-Compress			ESS-Tip-Compress			ESS-Compress		
			UST	MFC	Total	Core	MFC	Total	Core	MFC	Total
R. sphaeroides	0.2	0.4	0.1	0.1	1	0.1	0.0	1	0.2	0.0	1
Human RNA-seq	4.0	6.6	1.6	0.8	9	1.3	0.7	9	5.0	0.6	12
Gingiva metagenome	4.3	5.5	1.2	0.7	7	1.0	0.7	7	3.4	0.6	10
Soybean	5.7	9.6	0.8	0.6	11	0.7	0.7	11	2.4	0.5	13
Tongue metagenome	7.4	8.7	1.6	0.8	11	1.9	1.1	12	7.6	0.9	17
Whole human	95	106	11	7	124	10	6	122	40	7	152

The column for BOSS includes the time for $k$-mer counting the reads using KMC [21], the time to run BOSS construction, and the time to run LZMA. The total time in UST-Compress, ESS-Tip-Compress and ESS-Compress include the time to compute *cdBG* from the reads using BCALM. The time to compute *cdBG* is same for all three. The columns labelled *core* refer to Algorithm 1. ESS-Tip-Compress core uses the specific instance of Algorithm 1 defined in Sect. "ESS-Tip-Compress: a simpler alternative"

Note that MFC is one of many DNA sequence compressors that can be used with our algorithms. MFC is known to achieve superior compression ratios but is slower for compression/decompression than other competitors [38]. We recommend using MFC since it was not the time or memory bottleneck during compression, in our datasets.

## Discussion

In this paper, we presented a disk compression algorithm for $k$-mer sets called ESS-Compress. ESS-Compress is based on the strategy of representing a set of $k$-mers as a set of longer strings with as few total characters as possible. Once this string set is constructed, it is compressed using a generic nucleotide compressor such as MFC. On real data, ESS-Compress uses up to 42% less characters than the previous best algorithm UST-Compress. After MFC compression, ESS-Compress uses up to 27% less bits than UST-Compress.

We also presented a second algorithm ESS-Tip-Compress. It is simpler than ESS-Compress and does not achieve as good of compression sizes. However, the difference is less than 8% on our data, and it has the advantage of being about twice as fast and using significantly less memory during compression. For many users, this may be a desirable trade-off.

Our algorithms can also be used to compress information associated with the $k$-mers in *K*, such as their counts. Every $k$-mer in *K* corresponds to a unique location in the enriched string set. The counts can then be ordered sequentially, in the same order as the $k$-mers appear in the string set, and stored in a separate file. This file can then be compressed/decompressed separately using a generic compressor. After decompression of the enriched string set, the order of $k$-mers in the output SPSS will be the same as in the counts file.

We discussed several potential improvements to ESS-Compress, such as allowing more edges in the compacted de Bruijn graph to be absorbing or exploring the space of all path covers. We also gave a lower bound to what such improvements could achieve and showed they cannot gain more than 2% in space on our datasets. This makes these improvement of little interest, unless we encounter datasets where the gap is much larger.

ESS-Compress works by removing redundant $(k-1)$-mers from the string set, but a more general strategy could be to somehow remove $\ell$-mer duplicates, for all $\ell _{min} \le \ell \le k-1$. Such a strategy would require novel algorithms but would still be unable to reduce the characters per $k$-mer below one. On our datasets, this amounts to at most a 30% improvement in characters, which would be further reduced after MFC compression. It is also not clear if a 30% improvement in characters is even possible, since this kind of strategy would require a more sophisticated encoding scheme with more overhead.

Another direction to achieve lower compression sizes is to look beyond string set approaches. We observe, for example, that the large improvement of ESS-Compress compared to UST-Compress, measured in the weight of the string set, was significantly reduced when measured in bits after MFC compression. This indicates that some of the work done by ESS-Compress duplicates the work done by MFC on UST, which is itself designed to remove redundancy in the input. Thus, generic compressors such as MFC could potentially be modified to work directly on $k$-mer sets.

We believe that the biggest opportunity for improving the two algorithms of this paper are the compression time and memory. The time is dominated by the initial step of running BCALM2 to find unitigs. It may be possible to avoid this step by running UST directly on the non-compacted graph. Such an approach was taken in [28], and it would be interesting to see if it ends up improving on the memory and run-time of BCALM2. The memory usage, on the other hand, can likely be optimized with better software engineering. The current implementation of Algorithm 2 is done in a memoized bottom-up manner. Instead, a top down iterative implementation can reduce memory usage by directly writing to disk as soon as a vertex is processed. A “max-depth” option in Algorithm 2 could also be used to limit the depth of the recursion, thereby controlling memory at the cost of the compression ratio.

Another practical extension of ESS-compress is to allow the compression of associated information. Each $k$-mer, for example, could have an abundance. count associated with it. ESS-Compress representation defines an ordering on the $k$-mers. This ordering can be tracked through the algorithm and can then be used to sort the input associated data in the same order. Then, the associated data can be further compressed using LZMA (or any suitable compressor) and distributed with the ESS-Compress representation. The decompression algorithm would then similarly track the reordering of $k$-mers and apply the same permutation to the associated data.

## Footnotes

1 Though we did not explain it in [26], UST always returns normalized paths. It flips any vertex that is in the wrong orientation on its path, by reverse complementing its label, without affecting anything else.

2 Note that the vertices of this graph (i.e. *cdBG*(*K*)) correspond to maximal unitigs in the non-compacted graph (i.e. *dBG*(*K*)). We will generally use “vertex” and “unitig” interchangeably, to refer to a vertex in *cdBG*(*K*). We never use “unitig” to refer to a type of path in *cdBG*(*K*).

3 In our code, we actually allow a slightly broader definition of absorption. In particular, we also allow an edge to be absorbing if $u^p$ is an initiator and $s^p = 1$, or if $u^p$ is an initiator and $|lab(u^p)| \ge 2k - 2$. For the sake of simplicity, we do not consider this edge case in the paper.
